# Commercial Free-Range Laying Hens’ Preferences for Shelters with Different Sunlight Filtering Percentages

**DOI:** 10.3390/ani12030344

**Published:** 2022-01-31

**Authors:** Md Sohel Rana, Caroline Lee, Jim M. Lea, Dana L. M. Campbell

**Affiliations:** 1 Department of Animal Science, School of Environmental and Rural Science, University of New England, Armidale, NSW 2351, Australia; ranasoheldvm06@gmail.com; 2 Agriculture and Food, Commonwealth Scientific and Industrial Research Organisation (CSIRO), Armidale, NSW 2350, Australia; caroline.lee@csiro.au (C.L.); jim.lea@csiro.au (J.M.L.); 3 Department of Livestock Services, Ministry of Fisheries and Livestock, Dhaka 1215, Bangladesh

**Keywords:** Australia, chicken, free-range, preference, radiation, range enrichment, shelter, ultraviolet

## Abstract

**Simple Summary:**

Australian sunlight is intense, and may impact range use by free-range hens. Range design and management are important for optimising commercial layer farms where artificial shelters may offer protection for ranging hens. This study investigated preferences among 34–40-week-old hens for artificial shade cloth shelters of different densities, using two flocks on a commercial farm during the summer. Three types of sunlight-filtering shade cloth shelters, i.e., blocking 50%, 70%, and 90% of ultraviolet (UV) light, each with three replicates, were placed on the range for each flock. The number of hens under each shelter was counted at 30-min intervals using image snapshots from video recordings for 14 to 17 days. An on-site weather station recorded sunlight intensity across different spectra, ambient temperature, and relative humidity. During the day, hens generally preferred the 90%, followed by 70% and 50% sunlight-filtering shelters. However, fewer hens were observed underneath shelters during times of peak sun intensity. Shelter preferences were mostly impacted by ambient temperature in both flocks, with all sunlight spectra having different degrees of effect depending on the shelter type and flock. Overall, shelters comprising higher densities of sunlight-filtering artificial cloth were preferred by hens on the range, but these may not be sufficient to attract more hens outside during intense sunlight and hot climatic conditions.

**Abstract:**

Extreme sunlight might be aversive to free-range laying hens, discouraging them from going outside. Range enrichment with artificial shelters may protect hens from sunlight and increase range use. The preferences of 34–40-week-old Hy-Line Brown laying hens for artificial shelters were assessed by counting the number of hens under three densities of individual shelters (three replicates/density) from video recordings for 14 to 17 days for two flocks. The artificial shelters used shade cloth marketed as blocking 50%, 70%, and 90% of ultraviolet light, although other sunlight wavelengths were also reduced. Different sunlight spectral irradiances (ultraviolet radiation (UV_AB_) (288–432 nm), photosynthetically active radiation (PAR) (400–700 nm), and total solar radiation (TSR) (285 nm–3000 nm), ambient temperature, and relative humidity were recorded with an on-site weather station. There was a significant interaction between sunlight-filtering shelter and time of day (both Flocks, *p* < 0.0001), i.e., hens preferred shelters with the highest amount of sunlight-filtering at most time points. Regression models showed that the most variance in shelter use throughout the day resulted from the ambient temperature in both flocks, while sunlight parameters had different degrees of effect depending on the shelter type and flock. However, fewer hens under the shelters during the midday period suggest that during periods of intense sunlight, hens prefer to remain indoors, and artificial structures might not be sufficient to attract more hens outside.

## 1. Introduction

The important positive attributes of free-range (including organic) layer farming are the birds’ access to an outdoor range, exposure to natural daylight (sunlight), ability to move freely, increased space to better regulate social interactions, and opportunities for expression of natural behaviours [1,2]. Free-range systems may also have some potential risks such as parasitic infections [3], increased disease exposure [4], heat stress [5], and predation [6,7]. However, welfare benefits such as reduced plumage damage and reduced footpad dermatitis can be seen in individuals that range more [8,9]. The number of hens using the range in the first few weeks following the opening of the pop holes is typically low which gradually increases upon adaptation to the outdoor environment [10,11]. However, a range of external factors impact the daily outdoor range use of hens, even after acclimatisation, including weather conditions [12,13,14], season [15], time of day [16,17], and range enrichments [18,19]. Sometimes, hens may hesitate to venture outside or only use certain areas of the range closer to the pop holes [16,20,21,22]. The distribution of hens on the range can depend on range features such as vegetation and other enrichments [17,22]. A lower use of the outdoor range at the flock level may lead to increased feather and injurious pecking [23,24] or crowding of hens closer to the shed may cause smothering problems leading to bird mortality [25].

The outdoor range needs to be attractive to increase use by hens, i.e., offering different kinds of natural or artificial shelters and/or shades within the range [10,18,26]. These shelters may increase hens’ ranging by serving as protection from predators [27,28] or diffusing intense sunlight [17,29]. Laying hens might exhibit preferences for specific types of natural shelter options [30] as their ancestors were accustomed to dense vegetation. However, for range areas that may not have established vegetation, artificial shelters can provide protection to increase range usage and/or improve range use distribution. The exact features of these artificial shelters are likely to impact the extent to which hens use them [17,29] and the most preferred features need to be better understood.

Artificial shelters that provide protection from sunlight may be particularly important for free-range hens in climates with more extreme sunlight conditions such as those experienced in Australia during the summer months. The sunlight spectrum contains all forms of ultraviolet (UV) radiation: UVA (315–400 nm), UVB (280–315 nm), and UVC (100–280 nm), of which only UVA and UVB reach the earth’s surface. Hens can visually perceive UVA and UVB has a physiological effect on the synthesis of vitamin D_3_ in featherless skin [31]. However, high intensities and/or overexposure of UV radiation may have damaging effects [32,33], and, thus, hens might avoid direct sunlight at its most intense. Intense sunlight can also be visibly bright where the photosynthetically active radiation (PAR) wavelengths (400–700 nm) may be visually aversive and infrared wavelengths (>700 nm) are associated with heat. Different sunlight wavelengths as well as associated ambient climatic temperature and humidity variables may all impact the motivation of hens to use artificial shelters.

Behaviourally, studies in both free-range layers and meat chickens show that birds range more and are more active during the early morning and late afternoon periods compared to around midday [16,22,34]. Previous studies assessing shelter preferences on commercial farms within Australia have shown hens preferred higher density (% of UV filtering) shade cloth structures that filtered the most UV radiation [17] although preferences varied with time of day [29]. Hens also preferred artificial horizontal structures including those with one vertical side rather than vertical shelters alone [29]. However, shelter height, orientation, and cover density were all factors that affected hen preferences [29]. These studies to date highlight the complexity around optimal artificial shelter design. Further confirmation of hen preferences for artificial shelter cloth densities in relation to different sunlight wavelengths and ambient climatic variables is needed for optimising free-range systems in hot climates.

This study was conducted to assess the use by hens of different sunlight filtering shade cloth shelters in relation to different sunlight wavelengths on the range of a commercial free-range laying hen farm in Australia. The study hypothesised that hens would prefer the shelters that blocked a greater amount of sunlight, particularly when there was high sunlight intensity.

## 2. Materials and Methods

### 2.1. Animals and Husbandry

The study was conducted using two individual flocks (Flock-A, and Flock-B) at a single commercial free-range laying hen farm (comprised of multiple sheds and associated range areas) during the summer months (December 2020 to March 2021) in Queensland, Australia. Both flocks, comprising approximately 20,000 Hy-Line Brown laying hens each were studied from 34 to 40 weeks of age. The birds were from the same hatchery and reared indoors for 16 weeks (10–15 Lux) with the same resources, feed, and housing management as per the national laying hen guidelines [35] before shifting into the free-range facility. From 16 to 20 weeks, the hens were housed inside the indoor aviary with standard farm management protocols and resource access as per the national laying hen guidelines [35] and artificial lighting of approximately 70 Lux. At 20 weeks of age, hens were provided range access via pop holes (09:00–20:00 h). Hens were given 14 weeks of range acclimation before the study commenced.

### 2.2. Study Sites

**Figure 1:**
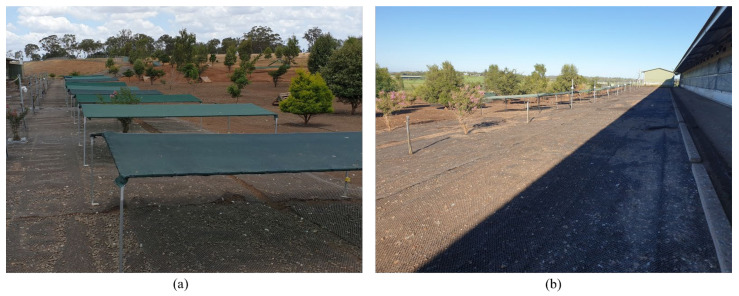
Experimental set-up in two different sheds of a commercial free-range farm: (**a**) Flock-A, and (**b**) Flock-B.

The two study sites within the larger farm property each had distinct land layout and vegetation within the range but identical resources inside the sheds and the same management practices. Both sites had an indoor shed, which was longitudinal in the east-west position with an outdoor range at both the north and south face. Hens within the shed could only access the range on either the north or south face due to an internal shed division, thus each shed actually contained 40,000 hens total. The south side of each shed was used for this study. The indoor sheds included an aviary system, furnished with feeders, drinkers, nest boxes and perches. Feed and water were provided *ad libitum* inside the shed only. The base of the shed sidewalls (0.62 m) was made from solid materials (poly panel) and the upper parts were covered by curtains up to the ceiling. The indoor shed temperature and relative humidity were maintained both mechanically by lowering and raising the curtains and automatically with fans throughout the study periods. When the curtains were raised (between 23 and 29 °C), sunlight could enter the barn, although the shed was positioned so this was minimised during the summer months. Each of the indoor sheds measured 120 L × 20 W × 8 m H with an indoor stocking density of 9 hens/m^2^. The outdoor stocking density was 1500 hens/ha (equivalent to 0.15 hens/m^2^). Pop-holes for range access were 0.55 m in height and located in the sidewalls. There was a total of 14 pop holes (6 m L × 0.62 m W) on each side, but typically, only half were opened for the full shed length. The range area adjacent to the shed wall (2.5 m length) was covered with compact gravel, then the immediate range area (12 m length) was covered with heavy weed fabric, followed by approximately 25 m length of uncovered (dirt) area, and the rest of the range was covered with grass. The total range area was approximately 13 hectares in size and thus the grassed area was expansive but typically few hens were observed in the farthest range areas (producer communication to DLMC, 2020). A number of trees were establishing within the range area, planted at varying distances from the shed past the gravel and fabric-covered areas (Figure 1). The boundaries of the range area were wire fences. During the observation periods, the daylight hours in the study sites were 04:57–18:51 h (at the beginning) and 05:14–18:54 h (at the end) for Flock-A, and 05:29–18:47 h (at the beginning) and 05:46–18:28 h (at the end) for Flock-B. The average minimum and maximum outdoor ambient temperatures in Flock-A were recorded as 24.1 ± 0.10 °C and 26.6 ± 0.10 °C respectively, and average relative humidity was 51.4 ± 0.27%; in Flock-B, the average minimum and maximum outdoor ambient temperatures were recorded as 24.0 ± 0.11 °C and 27.3 ± 0.11 °C respectively, and average relative humidity was 49.3 ± 0.17%.

### 2.3. Experimental Set-Up

To test the preferences of hens for shade cloth shelters of different densities, three types of shade cloth shelters with three replicates each were used: (i) 50% UV block (Coolaroo, 484866, Shade cloth, Rainforest), (ii) 70% UV block (Garden Shield, SC303610CG, HDPE, Cottage Green where supplier labelling indicated 30% UV block but controlled testing showed it was actually 70% UV block), and (iii) 90% UV block (Coolaroo, 486921, Shade cloth, Rainforest) (Figure 1).

**Figure 2:**
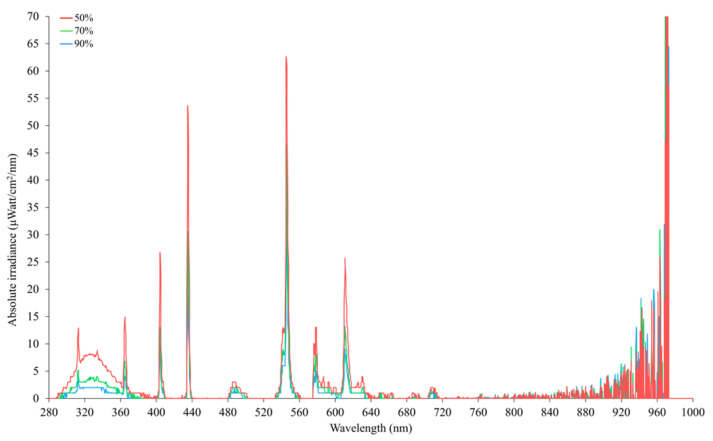
Spectral irradiance under different UV-filtering shade cloths (50%, 70%, and 90% UV block) as measured by an Ocean Insight Flame-S-XR1 Spectroradiometer at a distance of 20 cm with each type of shade cloth placed over a set of three Exo Terra^®^ (Rolf C. Hagen, Montreal, QC, Canada) pet reptile bulbs (Reptile UVB200, 25W, PT2341).

The UV filtering percentages of the treatment shelter cloths were confirmed using an Ocean Insight Flame-S-XR1 Spectroradiometer (200–1025 nm; Quark Photonics, Melbourne, VIC, Australia) set with an integration time of 180,000 μs and integration range from 280–1000 nm (Figure 2). Measurements were taken at a distance of 20 cm with each type of shade cloth placed over a set of three Exo Terra^®^ (Rolf C. Hagen, Montreal, QC, Canada) pet reptile bulbs (Reptile UVB200, 25W, PT2341) used as a standard, controlled source of UV radiation. Although the shade cloths are marketed as blocking UV radiation, they also filtered out solar radiation in the visible spectrum (Figure 2). Each shelter (4 m L × 3 m W × 1 m H) was positioned in a straight line parallel with the shed 10.5 m away from the pop holes. This distance was selected to avoid the shadow of the shed and to entice the hens farther out onto the range. Shelters were placed 3 m apart following the repeating pattern of 90%, 70%, and 50% UV block shade cloth in Flock-A and 70%, 90%, and 50% UV block shade cloth in Flock-B (Figure 1). The structure of the shelter was made of galvanised steel and shade cloth was stretched tight over the frame to minimise its movement in the wind with a small apex along the centre. Temperature and humidity loggers (Tinytag Plus 2, TGP-4500; Gemini Data Loggers Ltd., West Sussex, UK) were placed under each shelter on the rear left post at 300 mm height with automated logging at 15 min intervals. The position of these loggers resulted in them sometimes being shaded and sometimes being under direct sunlight, depending on the position of the sun. A high-resolution security camera system (Hikvision DS-7608NI-I2-8P CCTV NVR Recorder) was installed with a camera (Hikvision DS-2CD2355FWD-I2 CCTV 6MP Turret cameras) on a stand 1.6 m in front of each shelter to capture the entire shelter and the shadows that were cast during the day (Figure S1). Each IP camera was individually cabled back to a small enclosure mounted within the range that contained a Hikvision Ethernet POE Switch (Model DS-3e0109P-E(C)) that powered the cameras as well as a set of NanoBeam^®^–ac’s (model NBE-5AC-Gen2; Ubiquiti Inc., New York, NY, USA) that wirelessly routed the cameras back to the NVR system set up in the site office. An MEA weather station (Green Brain, 41 Vine Street, Magill, SA, Australia) was set-up on the respective farm site for recording sunlight and climatic variables and recorded weather data every 15 min over the study periods. The weather station was mounted on a post (user supplied) at a height of 1 m (height as instructed by the manufacturer) and included different sensors (UV3pAB UV sensor (288–432 nm), QS5 PAR pyranometer (400–700 nm), and SR-05 pyranometer (285–3000 nm)) for recording sunlight variables including ultraviolet radiation (UV_AB_) (W/m²), photosynthetically active radiation (PAR) (μmol/m^2^/s), and total solar radiation (TSR) (W/m^2^), respectively. The TSR included UV_AB_, PAR and infrared (IR) wavelengths and was used to extract IR (700 nm–3000 nm) (W/m²). Additionally, an air temperature and relative humidity sensor recorded the ambient temperature (°C), relative humidity (%), barometric pressure (mBar), dew point (°C), voltage (V), and vapor pressure deficit (kPa). As the study was primarily focused on the hen preferences for different shelters relative to sunlight variables, only solar radiation spectra, air temperature, and relative humidity data were considered in the final analyses.

### 2.4. Observations and Data Collection

The shelters were installed when the hens were 34 weeks of age with 2 weeks allowed for habituation to the range shelters before the study observations began. Recording was continuous during daylight hours for approximately 5 weeks for Flock-A and 4 weeks for Flock-B. Due to temporary failures in video recording, a total of 14 days videos for Flock-A and 17 days for Flock-B were analysed and these days were not consecutive within the recording period. For assessing shelter preferences, image snapshots from video records were taken at 30 min intervals from 30 min after pop hole opening (i.e., 09:30) until just before sunset (i.e., 18:30). The images were imported into Image-J 1.53a software (Wayne Rasband, National Institute of Health, MA, USA) and an observer counted the number of hens both under the individual shelters and on top of the shelters. When there were two observers, both researchers discussed the counts on common snapshots to ensure agreement. Observers were not blinded to the shade cloth densities given the differing darkness of the shadows cast by the shelters, but each observer conducted counts for all densities to minimise observer bias for a specific treatment density. On sunny days, the area for counting the hens under the shelter was defined by the shadow that the shelter cast (the exact position of the shadow varied throughout the day) (Figure S1). On cloudy days without a prominent shadow, the counting area was considered as the area directly underneath the shelter frame. If the individual hens could not be clearly identified due to crowding under the shelter, the number of hens was estimated in the group by counting the birds within a certain area and then multiplying that number by the counted area (this occurred on 41 occasions out of 5301 observations for both flocks).

### 2.5. Data and Statistical Analyses

All observations for each flock were analysed separately. A combined total of 5301 observations were made over the 14-day period in Flock-A (2394 observations), and 17-day period in Flock-B (2907 observations) to count both the hens underneath and on top of the shelters. The number of hens counted in each observation was matched with the corresponding weather parameters during the 15-min period directly prior to the observation time point. Weather parameters included the UV_AB_, PAR, TSR, ambient temperature and relative ambient humidity, and temperature and relative humidity readings from the loggers underneath the shelters. The hen count data contained a considerable number of ‘0’ values (when no hens were under or on top of the shelters) and were not normally distributed, thus these data were log (x + 1) transformed to include the ‘0’ values in the analyses as well as to approach data normality. To test the preferences of the hens to be underneath the shelters during the study period, data were analysed using JMP^®^ 14.0 (SAS Institute, Cary, NC, USA) with α level set at 0.05. General linear mixed models (GLMM) were applied with the different UV-filtering percentages, time of day, and their interaction included as fixed effects and shelter replicate nested within UV-filtering percentage as a random effect. A separate model with the same parameters was fitted to assess the preferences of the hens to be on top of the shelters. While the different sunlight filtering percentages were not predicted to affect hen preferences on top of the shelters, there may have been social influences if shelters with more hens underneath them, also had more hens located on top. The studentised model residuals were visually inspected for confirming homoscedasticity. Where significant differences were present, post hoc Student’s t-tests were applied to the least squares means with Bonferroni corrections to the α level to account for multiple post-hoc comparisons. The means of the temperature and relative humidity underneath the shelter were plotted, along with the mean ambient temperature and humidity during the day. However, these data were not statistically analysed as their positioning on the rear leg of the shelters resulted in the loggers sometimes being under direct sunlight which meant that they were not always an accurate measure of the temperature experienced by a hen when under the shaded part of the shelter.

To investigate the effects of sunlight variables on shelter use by hens during the day (presence under the shelter regardless of shelter type), an overall linear regression model was constructed for each flock using a summarised dataset where values within each UV-filtering percentage were averaged across all three replicates for each time point for each day (*n* = 798 per UV-filtering percentage in Flock-A, and *n* = 969 per UV-filtering percentage in Flock-B). Before setting the model, IR spectrum values were extracted from the TSR readings by subtracting UV_AB_ and PAR. A conversion value (μmol/m^2^/s to W/m^2^) as described by Thimijan and Heins [36], was applied to the PAR readings so all measures were in the same units for calculating the IR values. The number of hens underneath the UV-filtering shelters were included as the dependent variable, whereas sunlight variables (UV_AB_, PAR and IR), ambient temperature, and relative ambient humidity were included as independent variables in the model. Prior to running the model in R statistical software [37], the collinearity among the independent variables were checked through determination of variance inflation factors (VIF). Due to collinearity (VIF ≥ 10) among the sunlight variables, the ridge regression [38] was chosen to best fit the predictors into the model using the ‘lmridge’ package in R [39]. The relative contributions of the predictors in the regression model were estimated by the R package ‘relaimpo’ [40]. All independent variables were initially included in the model with nonsignificant variables (*p* ≥ 0.10) removed through backward elimination until the model of best fit was produced based on the adjusted R^2^ values. To specifically determine how sunlight and weather variables may affect the use of the different shelter types, individual linear ridge regression models were performed separately for each UV-filtering percentage with the number of hens underneath included as the dependent variable, and the sunlight variables (UV_AB_, PAR, and IR), ambient temperature, and relative ambient humidity included as independent variables. Nonsignificant variables (*p* ≥ 0.10*)* were removed through backward elimination. The raw values are plotted in the figures.

## 3. Results

### 3.1. Shelter Preferences

**Figure 3:**
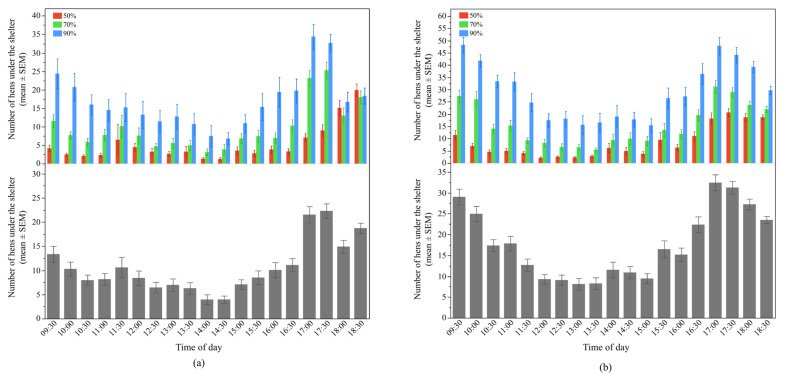
The mean (±SEM) number of hens underneath the shelters during the day: (**a**) Flock-A: with different UV-filtering percentages (50%, 70%, and 90%) (**top**), under all UV-filtering shelters (**bottom**); (**b**) Flock-B: with different UV-filtering percentages (50%, 70%, and 90%) (**top**), under all UV-filtering shelters (**bottom**). Note the different Y-axis scales between Flock-A and Flock-B. Raw values are presented with analyses conducted on transformed data.

There was a significant interaction between UV-filtering shelter and time of day for hen preferences in both Flock-A (F_36, 2331_ = 3.49, *p* < 0.0001*),* and Flock-B (F_36, 2844_ = 2.63, *p* < 0.0001) (Figure 3). In general, at most observation points throughout the day, more hens were seen under the 90% UV-filtering shelters in both flocks, but at some time points their preferences were similar for all filtering percentages (*p* > 0.001*)* (Figure 3).

Overall, more hens were found underneath the 90% UV-filtering shelters (LSM mean ± SEM, Flock-A: 16.9 ± 2.67 hens; Flock-B: 29.1 ± 1.52 hens), followed by the 70% (LSM mean ± SEM, Flock-A: 9.7 ± 2.67 hens; Flock-B: 15.7 ± 1.52 hens) then 50% UV-filtering shelters (LSM mean ± SEM, Flock-A: 5.2 ± 2.67 hens; Flock-B: 8.4 ± 1.52 hens) in both study flocks (Flock-A: F_2, 6_ = 16.25, *p* = 0.004, and Flock-B: F_2, 6_ = 134.09, *p* < 0.0001). The use of the shelter shade by hens varied throughout the day in both Flock-A (F_18, 2331_ = 44.64, *p* < 0.0001) and Flock-B (F_18, 2844_ = 75.11, *p* < 0.0001), with peaks in the morning and in the late afternoon, compared to the midday (*p* < 0.003) (Figure 3).

**Figure 4:**
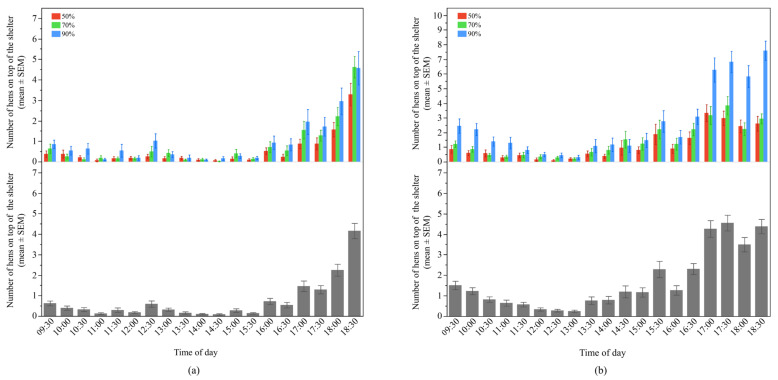
The mean (±SEM) number of hens on top of the shelters throughout the day: (**a**) Flock-A: with different UV-filtering percentages (50%, 70%, and 90%) (**top**), on all UV-filtering shelters (**bottom**); (**b**) Flock-B: with different UV-filtering percentages (50%, 70%, and 90%) (**top**), on all UV-filtering shelters (**bottom**). Note the different Y-axis scales between Flock-A and Flock-B for the top graphs. Raw values are presented with analyses conducted on transformed data.

In contrast, there was no significant interaction between UV-filtering shelter and time of day for the number of hens on top of the shelters in Flock-A (F_36, 2331_ = 0.89, *p* = 0.65); whereas a significant interaction was found in Flock-B (F_36, 2844_ = 2.68, *p* < 0.0001) (Figure 4). In Flock-B, throughout the day, there was a general pattern of more hens on top of the 90% UV-filtering shelters in the morning and late afternoon relative to both the 50% and 70% shelters (*p* > 0.001) (Figure 4).

Overall, there was no difference for the number of hens on top of the shelters between the different UV-filtering percentages in Flock-A (LSM mean ± SEM, 50%: 0.52 ± 0.18, 70%: 0.75 ± 0.18, 90%: 0.96 ± 0.18, F_2, 6_ = 1.37, *p* = 0.32), but the time of day had an effect on the number of hens throughout the day (F_18, 2331_ = 41.78, *p* < 0.0001), with a gradually increasing trend after 17:00 compared to the rest of the day (*p* < 0.003) (Figure 4). In Flock-B, more hens were found on top of the 90% UV-filtering shelter with no differences between the 50% and 70% shelters (LSM mean ± SEM, 50%: 1.14 ± 0.24, 70%: 1.38 ± 0.24, 90%: 2.54 ± 0.24, F_2, 6_ = 9.14, *p* = 0.02). Time of day had an effect on the number of hens on top of the shelters (F_18, 2844_ = 36.56, *p* < 0.0001) with more hens observed in the late afternoon (*p* < 0.003) (Figure 4).

**Figure 5:**
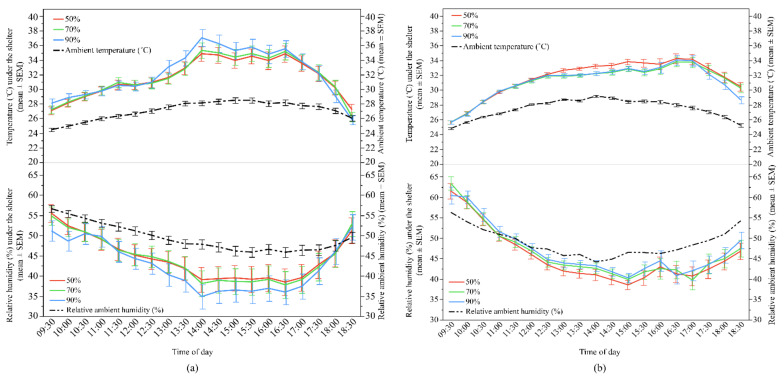
The mean (±SEM) temperature and relative humidity throughout the day underneath the shelters of different UV-filtering percentages (50%, 70%, and 90%) and ambient temperature and relative humidity: (**a**) Flock-A: temperature under the shelters and ambient temperature (**top**), relative humidity under the shelters and relative ambient humidity (**bottom**); (**b**) Flock-B: temperature under the shelters and ambient temperature (**top**), relative humidity under the shelters and relative ambient humidity (**bottom**). Raw values are presented with analyses conducted on transformed data.

The temperature and humidity loggers underneath the shelters were intended to provide measurements on ambient conditions the hens may have been experiencing. However, the placement of loggers at hen eye height on one of the rear posts of the shelters resulted in the loggers sometimes being under direct sunlight and sometimes being under the shelter shade. Figure 5 displays the temperature and relative humidity readings under each shelter type relative to the ambient temperature and relative ambient humidity readings obtained from the weather station which was placed 1 m above ground. The temperature under the shelters was higher than the ambient temperature, whereas relative humidity was lower than the relative ambient humidity during the daytime (Figure 5). The temperatures and relative humidity under the different shelter types were visually similar, but these data were not statistically analysed, as the loggers did not capture data as originally intended.

### 3.2. Sunlight Effects

A ridge regression model for each flock was performed to investigate the relationship between the number of hens underneath the shelters across all the UV-filtering percentages and the sunlight variables, ambient temperature and relative ambient humidity. The best-fit model results are presented in Table 1. In Flock-A, the model accounted for 34.21% of the variance in the use of all the UV-filtering shelters throughout the day. The ambient temperature, UV_AB_, IR, and relative ambient humidity contributed significantly to the model (F_3.35, 794.24_ = 120.50, *p* < 0.0001). However, all these predictors had a negative correlation with the number of hens under the shelters throughout the day (Table 1).

In Flock-B, the model accounted for 35.77% of the variance in the number of hens under the shelters with respect to sunlight and weather variables considered within the model. The majority of the variance was explained by the ambient temperature (49.01%), however IR, UV_AB_ and PAR also significantly contributed to the model (F_2.68, 965.98_ = 146.64, *p* < 0.0001, Table 1). The ambient temperature, UV_AB_, and IR were negatively correlated, and PAR was positively correlated with the number of hens underneath the shelters (Table 1).

The separate ridge regression models for each UV-filtering percentage showed differences in the relative impacts of the sunlight and weather variables on the number of hens underneath the shelters. For the 50%, 70%, and 90% UV-filtering shelter preferences, both sunlight and weather variables accounted for 51.71% (Flock-A: F_2.79, 263.03_ = 108.58, *p* < 0.0001) and 57.94% (Flock-B: F_2.53, 320.19_ = 156.77, *p* < 0.0001) of the variance for the 50% shelters, 40.35% (Flock-A: F_2.79, 263.03_ = 71.33, *p* < 0.0001) and 44.29% (Flock-B: F_2.68, 319.98_ = 71.26, *p* < 0.0001) of the variance for the 70% shelters, and 35.16% (Flock-A: F_3.08, 262.54_ = 51.13, *p* < 0.0001) and 37.77% of the variance (Flock-B: F_2.68, 319.98_ = 56.45, *p* < 0.0001) for the 90% shelters (Table 2).

**Figure 6:**
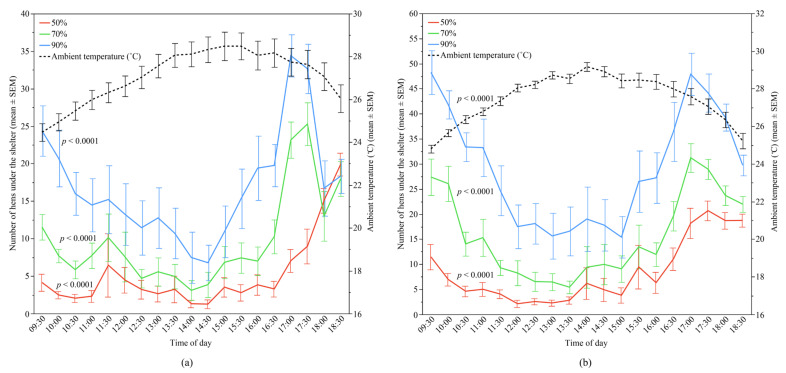
The mean (±SEM) number of hens under the different UV-filtering shelters (50%, 70%, and 90%) and the mean (±SEM) ambient temperature throughout the day: (**a**) Flock-A; (**b**) Flock-B (*p* > 0.10 indicates the variable had no significant effect and was removed from final model). Note the different Y-axis scales between Flock-A and Flock-B. Raw values are presented with analyses conducted on transformed data.

The ambient temperature significantly affected the preferences of the hens for each shelter type in both flocks (Flock-A: all *p* < 0.0001; Flock-B: all *p* < 0.0001) (Figure 6); this parameter was the greatest contributing factor for use of the 70% and 90% UV-filtering shelters. Temperature accounted for 38.52% and 40.75% of the variation in Flock-A for the 70% and 90% shelters respectively, and 45.57% and 71.58% of the variation in Flock-B for the 70% and 90% shelters, respectively. The results indicated that increased ambient temperature resulted in fewer hens under the shelters (Table 2).

**Figure 7:**
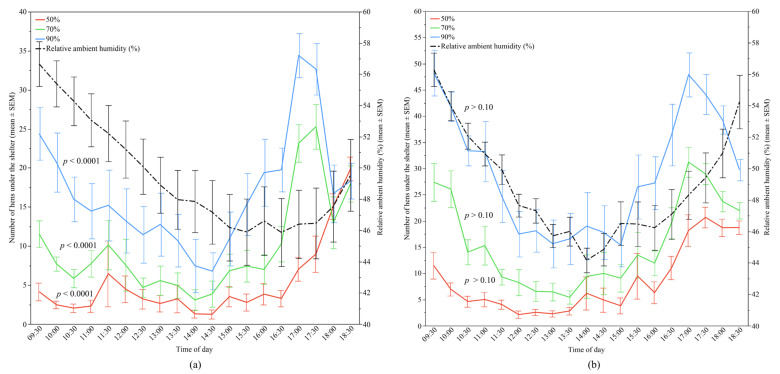
The mean (±SEM) number of hens under the different UV-filtering shelters (50%, 70%, and 90%) and the mean (±SEM) relative ambient humidity throughout the day: (**a**) Flock-A; (**b**) Flock-B (*p* > 0.10 indicates the variable had no significant effect and was removed from final model). Note the different Y-axis scales between Flock-A and Flock-B. Raw values are presented with analyses conducted on transformed data.

The relative ambient humidity also significantly contributed to the preferences of the hens for each shelter type in Flock-A (all *p* < 0.0001), but did not show an effect in Flock-B (Figure 7). However, in Flock-A, the relative contribution of the relative ambient humidity was less than 20% in the models of 50%, 70% and 90% UV-filtering shelters (accounting for 10.52%, 15.64%, and 18.85% of the variation, respectively), and had a negative correlation with the number of hens under the shelters (Table 2).

**Figure 8:**
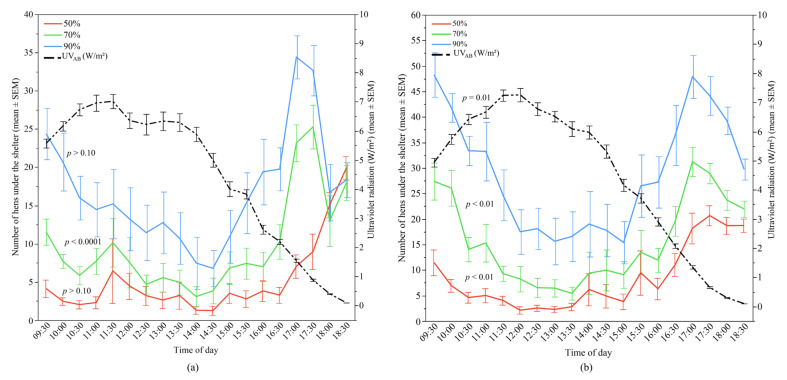
The mean (±SEM) number of hens under the different UV-filtering shelters (50%, 70%, and 90%) and the mean (±SEM) ultraviolet (UV_AB_) radiation throughout the day: (**a**) Flock-A; (**b**) Flock-B (*p* > 0.10 indicates the variable had no significant effect and was removed from final model). Note the different Y-axis scales between Flock-A and Flock-B. Raw values are presented with analyses conducted on transformed data.

UV_AB_ radiation only had a significant effect for the 70% UV-filtering shelter preferences (*p* < 0.0001) in Flock-A where it was the most contributory effect (45.57% variation) in that specific model (Figure 8). In contrast, UV_AB_ radiation showed a significant relationship with the use of all shelter types in Flock-B (all *p* ≤ 0.01) (Figure 8). The relative contribution of UV_AB_ among the predictors in Flock-B for 50%, 70%, and 90% UV-filtering shelter was 32.43%, 18.10%, and 9.20%, respectively, with the number of hens under the shelter decreasing with increasing UV_AB_ radiation (Table 2).

**Figure 9:**
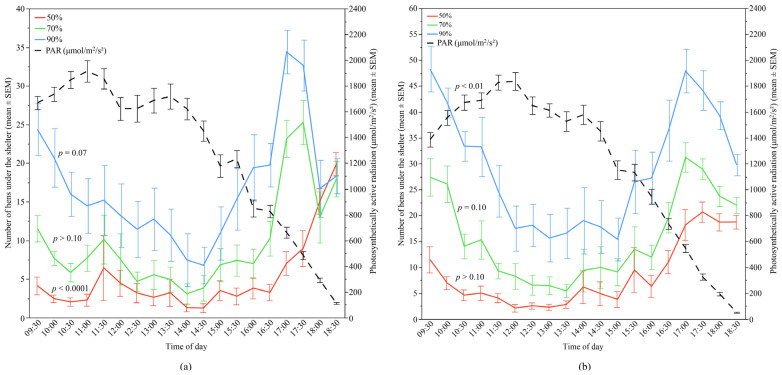
The mean (±SEM) number of hens under the different UV-filtering shelters (50%, 70%, and 90%) and the mean (±SEM) photosynthetically active radiation (PAR) throughout the day: (**a**) Flock-A; (**b**) Flock-B (*p* > 0.10 indicates the variable had no significant effect and was removed from final model). Note the different Y-axis scales between Flock-A and Flock-B. Raw values are presented with analyses conducted on transformed data.

In Flock-A, PAR had a significant negative correlation with the use by the hens of the 50% UV-filtering shelter (*p* < 0.0001) showing the greatest contributory effect (64.86% variation, Table 2) in the model, and a positive trend for the 90% shelters (*p* = 0.07) but no association with use of the 70% UV-filtering shelters (Figure 9). Whereas, in Flock-B, PAR was a significant contributing variable for use by the hens of the 90% UV-filtering shelters (*p* < 0.01), and it had a trend effect for the 70% shelters (*p* = 0.10), but no significant contribution for the 50% UV-filtering shelters (Figure 9). While the relative weight of PAR in the models of 90% and 70% UV-filtering shelter preferences was 9.58% and 17.91%, respectively, this had a positive relationship with the number of hens under the respective shelters, indicating increases in PAR also increased shelter use by the hens (Table 2).

**Figure 10:**
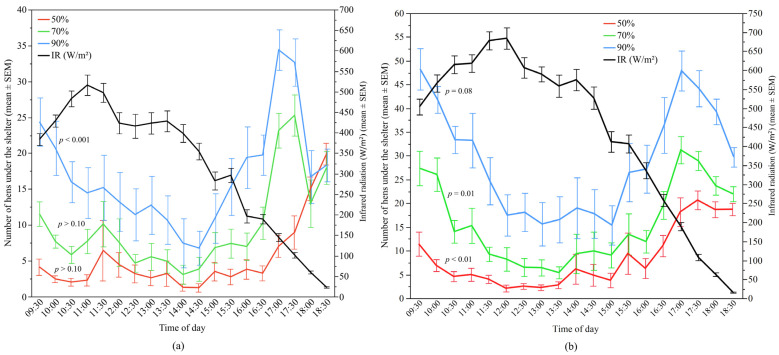
The mean (±SEM) number of hens under the different UV-filtering shelters (50%, 70%, and 90%) and the mean (±SEM) infrared radiation (IR) throughout the day: (**a**) Flock-A; (**b**) Flock-B (*p* > 0.10 indicates the variable had no significant effect and was removed from final model). Note the different Y-axis scales between Flock-A and Flock-B. Raw values are presented with analyses conducted on transformed data.

IR significantly affected shelter use of only the 90% UV-filtering shelters (*p* < 0.0001) in Flock-A. However, in Flock-B, IR significantly influenced shelter use of both the 70% and 50% UV-filtering shelters (both *p* ≤ 0.01) and had a trend of an effect for the 90% UV-filtering shelters (*p* = 0.08) (Figure 10). However, a negative correlation between IR and use of shelters indicated that the number of hens under the shelters decreased when IR increased (Table 2).

## 4. Discussion

This study assessed the use by hens of different sunlight filtering shade cloth shelters on the range of a commercial farm in Australia. Different sunlight wavelengths were also measured directly on-farm to determine if shelter preferences were dependent on ambient conditions. The results showed that hens had clear preferences for shelters with the highest density, i.e., those that blocked the greatest amount of sunlight. However, relationships with temperature, humidity, and sunlight wavelengths were generally negative, with fewer hens under the shelters as the values of the weather parameters increased. This may have been a result of reduced ranging at times of peak sun intensity, which is consistent with the findings of previous studies.

Previous studies have shown that outdoor range enrichments will increase range use by hens and improve their distribution outside [10,18,19,27]. However, the effects of these enrichments can vary depending on the structural design, i.e., type (artificial/natural), location, height, orientation, and density [16,17,29,30]. Similar to other studies that have been conducted on commercial farms within Australia [17,29], the hens in our study showed clear preferences for the higher densities of the shade cloth with a linear relationship between use of the shelter and percentage of sunlight it filtered. These results confirm that hens can differentiate between shaded environments and will preferentially select the environment that provides the greatest amount of shaded protection. Anecdotal observations in the current study indicated that hens were sometimes crowded under the shade provided by trees on the range just beyond the artificial shelters, while shelters were comparatively empty. Formal counts were not made on this, as the camera position did not enable clear observations of hen numbers under the trees. This result aligns with the jungle fowl origins of domestic chickens, as well as previous observations that the greatest numbers of hens on commercial farms are attracted to natural shelter provided by trees [10], or will preferably gather under dense vegetation and established trees [30]. While hens may not always seek shelter on the range and may use the sunlight for sunbathing [41] and warmth [12], conditions of extreme heat and intense sunlight are likely more aversive than enticing. Temperatures were not taken under the trees on the range in this study, but they may have provided a cooler environment than under the shelters as a result of blocking more sunlight, as well as evaporative cooling from transpiration. While the temperatures were recorded as hotter under the shelters than the ambient temperature, the temperature loggers were sometimes in direct sunlight, and thus, we are limited in the conclusions we can draw from these results.

Environmental factors that explained the number of hens under the shelters were ambient temperature, UV_AB_, and IR in both flocks, indicating that the overall use of shelters decreased with increasing intensity of these factors. The predominant influencing factor was ambient outdoor temperature. These results are in contrast to what was predicted, given that previous studies have shown that hens increase their use of shaded areas as temperatures increase, with few birds in nonshaded areas during the summer [18]. Richards et al. [12] reported that the percentage of hens ranging gradually decreased as temperatures increased above 17 °C, although the study was conducted in the UK, where much lower temperatures overall are experienced than those in the current study. Slow-growing broilers will increase their use of shelters as solar radiation increases [42,43], which is expected, given the damaging impact of UV light [32,33] even though UV radiation was not found to be a predictor of range use in fast-growing broilers [34]. The hens in this study showed variation in shelter use throughout the day, regardless of shelter type, corresponding with typical patterns of range use reported in other studies [12,15,19], including observations in different flocks of the same farm as the current study [44]. Thus, the negative relationships between the environmental predictor variables were likely a reflection of fewer hens on the range during peak sun periods. General range use was not measured in this study, but the observed patterns of shelter use suggest that in regions of intense sunlight such as many regions of Australia, hens prefer to remain inside during the midday period, regardless of the presence or absence of artificial shelters on the range. Further studies could assess if more trees [10], different designs of artificial shelters [29] or additional range enrichments [18,26] could entice hens outside. If temperature is a key variable affecting the shelter preferences of hens, then shelter size may be another variable to consider, as well as the extent to which temperature varies under the edges or centre of the shelters. Alternatively, remaining inside the shed could prevent heat stress; in some regions of Australia, range use will be prevented on days of high heat to prevent bird mortality [44]. Further study assessing temperatures under different shelter types, established trees, shrubs, and inside sheds will confirm the different microclimates which exist in a free-range system and how they affect hen locational preferences.

The positive relationships seen between shelter use and the PAR wavelengths demonstrates that hens were using shelters to avoid bright light. This may have been comfort-related, i.e., the same way humans will wear sunglasses or could be motivated to seek cover rather than being exposed to bright light. The 90% filtering shelter would have reduced the visibility of hens from above to a greater degree, and hens may have used it as protection from aerial predators [26,28]. This could also explain the higher use of shelters in the late afternoon, when sunlight wavelengths greatly decreased in intensity, but hens may still have been seeking protection from aerial predators. In contrast to this, hens also increased in numbers on top of the shelters in the late afternoon/evening, but this may have been related to a nighttime desire to roost [45]. While hens were kept inside the shed overnight, the setting sun may have stimulated motivation to seek elevation for those hens still ranging as sunset approached. These observations, in conjunction with environmental parameters only accounting for part of the variation in shelter use, indicate the interplay of many factors regarding a hen’s decision to reside under a shelter versus in the open range area.

## 5. Conclusions

This study found that higher densities of sunlight-filtering artificial cloth shelters are preferred by hens, and that temperature is a key variable affecting shelter use. All wavelengths of sunlight had some effect on shelter use, but the effect varied among the shelter densities and flocks in this study. The low use of all shelters during the midday period and negative relationships with temperature, humidity, UV_AB_ and IR suggest that the shelters may not be sufficient for attracting more hens to the range in periods of intense sunlight and hot temperatures, during which hens are typically observed to range less. Range enrichments of both artificial and natural shelters may encourage more hens outside. In the absence of established trees on the range providing a larger canopy cover and reduced temperatures underneath, cooler conditions inside the shed may be preferable, but further research is needed to confirm this.

## Figures and Tables

**Table 1:** Two ridge regression analyses (ridge parameter, k = 0.02) on the number of hens under the shelter throughout the day. Only variables that significantly contributed to the most parsimonious model are presented.

Flock	Predictor ^1^	β- Coefficient (Standardised) ^‡^	t-Value	*p*-Value	Adjusted R^2^ and Model’s F-Statistics	Relative Weight of the Predictors in the Model
Flock-A	Ambient temperature	−0.70	−12.61	<0.0001	R^2^-adjusted = 0.34	33.54%
	Relative ambient humidity	−0.46	−8.29	<0.0001	F_3.35, 794.24_ = 120.50, *p* < 0.0001	13.86%
	UV_AB_	−0.15	−2.17	0.03		25.36%
	IR	−0.24	−3.37	0.001		27.24%
Flock-B	Ambient temperature	−0.41	−15.90	<0.0001	R^2^-adjusted = 0.36	49.01%
	UV_AB_	−0.27	−3.78	<0.001	F_2.68, 965.98_ = 146.64, *p* < 0.0001	16.88%
	PAR	0.10	2.07	0.04		16.84%
	IR	−0.19	−3.70	<0.001		17.28%

^‡^ β-coefficients (standardised) of the predictor variables were estimated separately using the ridge regression coefficient in ‘R’ as the original ridge package did not include the ‘β-coefficient’ value in the regression outputs. ^1^ UV_AB_ (ultraviolet radiation A and B wavelengths), PAR (photosynthetically active radiation), IR (infrared radiation).

**Table 2:** Multiple ridge regression analyses (ridge parameter, k = 0.02) on the number of hens under different UV-filtering shelters throughout the day. Only variables that significantly contributed to the most parsimonious model are presented.

UV-Filtering Shelter	Flock	Predictor ^1^	Β-Coefficient (Standardised) ^‡^	*t*-Value	*p*-Value	Adjusted R^2^ and Model’s F-Statistics	Relative Weight of the Predictors in the Model
50%	A	Ambient temperature	−0.59	−7.16	<0.0001	R^2^-adjusted = 0.52	24.62%
		Relative ambient humidity	−0.36	−4.42	<0.0001	F_2.79, 263.03_ = 108.58, *p* < 0.0001	10.52%
		PAR	−0.56	−13.25	<0.0001		64.86%
	B	Ambient temperature	−0.40	−11.24	<0.0001	R^2^-adjusted = 0.58	34.26%
		UV_AB_	−0.29	−3.18	<0.01	F_2.53, 320.19_ = 156.77, *p* < 0.0001	32.43%
		IR	−0.28	−3.08	<0.01		33.31%
70%	A	Ambient temperature	−0.77	−8.48	<0.0001	R^2^-adjusted = 0.40	38.52%
		Relative ambient humidity	−0.46	−5.11	<0.0001	F_2.79, 263.03_ = 71.33, *p* < 0.0001	15.64%
		UV_AB_	−0.41	−8.87	<0.0001		45.85%
	B	Ambient temperature	−0.44	−10.63	<0.0001	R^2^-adjusted = 0.44	45.57%
		UV_AB_	−0.33	−2.85	<0.01	F_2.68, 319.98_ = 71.26, *p* < 0.0001	18.10%
		PAR	0.13	1.66	0.10		17.91%
		IR	−0.22	−2.70	0.01		18.42%
90%	A	Ambient temperature	−0.93	−9.99	<0.0001	R^2^-adjusted = 0.35	40.75%
		Relative ambient humidity	−0.68	−7.44	<0.0001	F_3.08, 262.54_ = 51.13, *p* < 0.0001	18.85%
		PAR	0.20	1.84	0.07		19.14%
		IR	−0.53	−4.92	<0.0001		21.26%
	B	Ambient temperature	−0.54	−12.28	<0.0001	R^2^-adjusted = 0.38	71.58%
		UV_AB_	−0.31	−2.51	0.01	F_2.68, 319.98_ = 56.45, *p* < 0.0001	9.20%
		PAR	0.24	2.85	<0.01		9.58%
		IR	−0.15	−1.78	0.08		9.63%

^‡^ β-coefficients (standardised) of the predictor variables were estimated separately using the ridge regression coefficient in ‘R’ as the original ridge package did not include the ‘β-coefficient’ value in the regression outputs. ^1^ UV_AB_ (ultraviolet radiation A and B wavelengths), PAR (photosynthetically active radiation), IR (infrared radiation).

## Data Availability

Data that support this study will be made available upon any reasonable request to the corresponding author.

## Supplementary Materials

The following supporting information can be downloaded at: https://www.mdpi.com/article/10.3390/ani12030344/s1, Figure S1: Snapshots of one of the 90% UV-filtering shelters in Flock-B showing use of the shelter and the immediate surrounding range area across one day.
