# Supplementary material for: Commercial Free-Range Laying Hens’ Preferences for Shelters with Different Sunlight Filtering Percentages
Source: Animals (Basel). 2022 Jan 31;12(3):344. doi: 10.3390/ani12030344 (PMC8833566; doi:10.3390/ani12030344)
Supplement: Supplementary file 1 [file animals-12-00344-s001.zip › animals-1517616-supplementary.pdf]

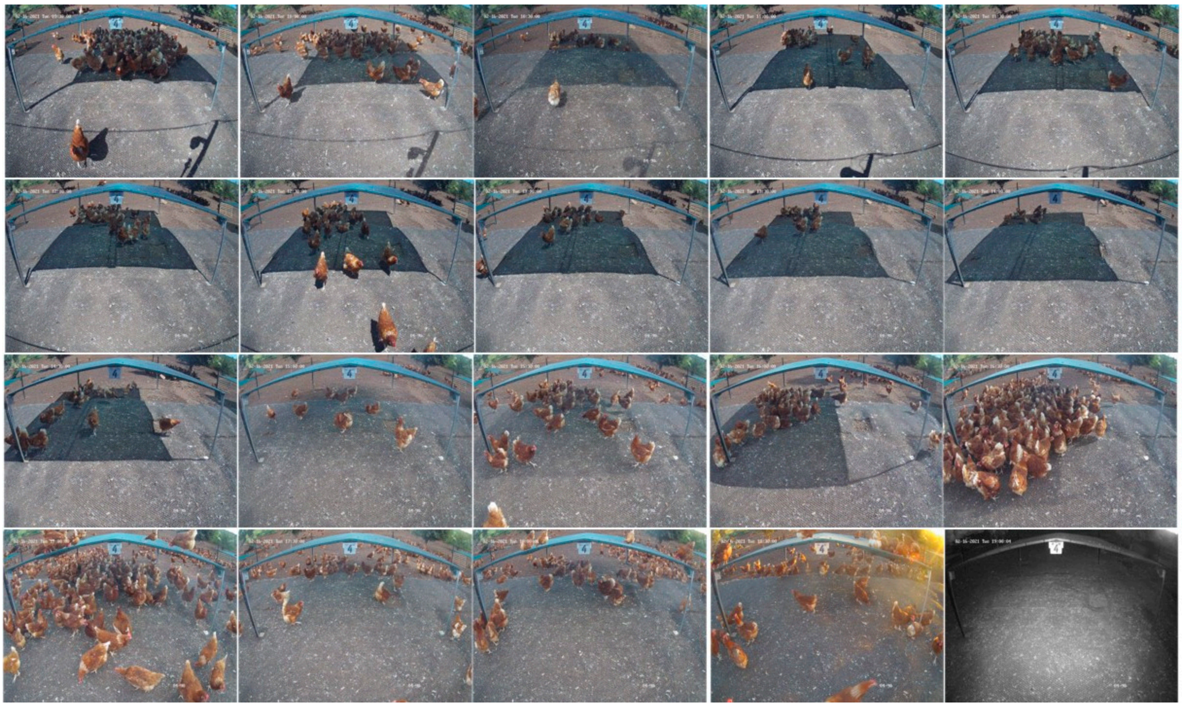

Figure S1. Snapshots of one of the 90% UV-filtering shelters in Flock-B showing use of the shelter and the immediate surrounding range area across one day.
